# Effects of a therapeutic exercise program in children with non-cystic fibrosis bronchiectasis: A pilot randomized controlled trial

**DOI:** 10.3389/fped.2022.953429

**Published:** 2022-09-15

**Authors:** Barbara Joschtel, Sjaan R. Gomersall, Sean Tweedy, Helen Petsky, Anne B. Chang, Stewart G. Trost

**Affiliations:** ^1^School of Human Movement and Nutrition Sciences, The University of Queensland, Brisbane, QLD, Australia; ^2^School of Health and Rehabilitation Sciences, The University of Queensland, Brisbane, QLD, Australia; ^3^Faculty of Health, Centre for Children’s Health Research, Queensland University of Technology, Brisbane, QLD, Australia; ^4^Department of Respiratory and Sleep Medicine, Queensland Children’s Hospital, Children’s Health Queensland, Brisbane, QLD, Australia; ^5^Child Health Division, Menzies School of Health Research, Charles Darwin University, Tiwi, NT, Australia

**Keywords:** physical activity, pediatrics, clinical trial, bronchiectasis, fundamental movement skills, respiratory disease, motor competence, fitness

## Abstract

**Background:**

In the absence of randomized controlled trials (RCTs) on the benefits of therapeutic exercise programs involving children with bronchiectasis, we undertook a pilot RCT to evaluate the effects of a play-based therapeutic exercise program on fundamental movement skill (FMS) proficiency. The effects of the program on cardiorespiratory fitness, perceived competence, and health-related quality of life (HR-QoL) were examined as secondary outcomes.

**Methods:**

Children [median (IQR) age: 6.8 (5.3–8.8) years] with bronchiectasis unrelated to cystic fibrosis were randomized to a 7-week therapeutic exercise program (*n* = 11) or wait-list control (*n* = 10). The exercise program comprised 7 × 60-min weekly sessions and was supplemented by a home-based program 2-days/week. Participants were assessed on: FMS (locomotor and object control skills) using the Test of Gross Motor Development 2nd Edition (TGMD-2); cardiovascular fitness by calculating the percent change in heart rate (%ΔHR) from rest to completion of the first stage of a submaximal treadmill test; perceived competence using Harter’s athletic competence subscale; and QoL with the PedsQL.

**Results:**

Significant group by time interactions were observed for locomotor and object control skills. Children completing the therapeutic exercise program exhibited significant improvements in both locomotor (pre 29.0 ± 2.0, post 35.2 ± 2.2, *p* = 0.01) and object control (pre 27.0 ± 2.0, post 35.5 ± 2.2, *p* = 0.01) skills, with no significant change in controls (pre 31.6 ± 2.1, post 31.8 ± 2.3 and pre 31.0 ± 2.1, post 32.3 ± 2.3, respectively). Among children completing the program, %ΔHR declined by 6% points, while %ΔHR declined only marginally among controls (0.9% points), but the group by time interaction was not statistically significant. The program had a small positive impact on competence perceptions (Cohen’s *d* = 0.2) and HR-QoL (Cohen’s *d* = 0.3).

**Conclusion:**

This pilot RCT provides preliminary evidence for the efficacy of a play-based therapeutic exercise program to improve proficiency in FMS and fitness in children with bronchiectasis. The results are sufficiently positive to warrant conducting a larger RCT testing the efficacy of the exercise program in children with bronchiectasis and/or other chronic respiratory conditions.

## Introduction

Globally bronchiectasis is recognized as a major cause of respiratory morbidity, mortality and health-care utilization (1). It is part of the chronic suppurative lung disease spectrum and caused by chronic airway inflammation resulting in abnormal dilatation of the bronchial wall (1, 2). Typical clinical features are recurrent or persistent wet cough (2, 3) and they regularly experience exacerbations of their condition resulting in frequent acute visits to the doctor, hospitalization, further deterioration of lung tissue, and decreased quality of life (QoL) (4, 5). The prevalence of bronchiectasis ranges from 0.2 to 15 cases per 100,000 children (2). Of particular concern, the prevalence is substantially higher among socially disadvantaged populations such as the indigenous communities of Australia, New Zealand, Pacific Islands, Alaska, and Canada (2, 3). For example, a study of indigenous children residing in central Australia reported a prevalence of 1,470 cases per 100,000 (2, 6).

Current guidelines for the treatment and management of bronchiectasis call for regular exercise as a means of improving cardiovascular fitness and QoL (3, 7). However, to date, the health benefits resulting from therapeutic exercise have not been systematically investigated in children with bronchiectasis. A recent study involving 39 children and adolescents with non-CF bronchiectasis reported active video gaming to improve aerobic fitness and muscular strength; however, because exercise was combined with an extensive home-based physiotherapy program comprising ventilatory muscle training and postural drainage with percussion, the independent effects of exercise training on the fitness and respiratory outcomes could not be delineated (8). We have previously shown that children with bronchiectasis are insufficiently active for health benefit (9) and exhibit significant developmental delays in fundamental movement skill (FMS) proficiency (10). Further, children with bronchiectasis who achieve their age equivalency for FMS have higher levels of physical activity (10). On the basis of these findings, and the consistent evidence that exercise training improves cardiorespiratory fitness and QoL in children with other respiratory conditions (11), we hypothesized that a play-based, therapeutic exercise program designed to improve FMS would be beneficial to the health and well-being of children with bronchiectasis. Therefore, the primary aim of this pilot randomized controlled trial (RCT) was to evaluate the efficacy of a play-based, therapeutic exercise program to increase proficiency in FMS in children with bronchiectasis. A secondary aim was to determine the effects of the program on cardiorespiratory fitness, perceived movement competence, and QoL.

## Materials and methods

### Participant recruitment

Children meeting the following inclusion criteria were eligible to participate in the study: diagnosed with bronchiectasis, aged between 4 and 13 years, and able to complete an exercise program. Bronchiectasis was diagnosed according to the then current guideline published by the Thoracic Society of Australia and New Zealand (3). Exclusion criteria were unstable medical condition, unstable emotional or behavioral status, or recent musculoskeletal injury (e.g., sprains, fracture, and muscle strains).

Participants were recruited through the Department of Respiratory and Sleep Medicine at the Royal Children’s Hospital (now called Queensland Children’s Hospital), Brisbane, Australia, between March 2015 and February 2017. Clinicians were provided a detailed study description, along with the inclusion and exclusion criteria, and asked to identify children who met the criteria and would benefit from participating in a therapeutic exercise program addressing movement skills and aerobic fitness. Parents of potential participants were subsequently contacted by the primary investigator (BJ) who formally assessed eligibility and provided details about the study. After discussing the study, parents with children who were eligible and interested in participating provided written informed consent. Children aged ≥7 years also provided written assent. Following established procedures for estimating the sample size for a pilot RCT (12), the minimum sample size requirement was set to 10 participants per treatment arm (*N* = 20). This calculation was based on an anticipated between-group difference of at least 0.8 standard deviations (SDs) for locomotor and object control movement skills, respectively (13). Ethical approval for this study was received from the Human Research Ethics Committee at the Children’s Health Queensland Hospital and Health Service (HREC/14/QRCH/136) and the Human Research Ethics Committee at the University of Queensland (2014001176). The RCT was prospectively registered with the Australian New Zealand Clinical Trials Registry (ACTRN12614000920695).

### Study design and setting

A single-site, open RCT was conducted in a clinical setting at a public hospital in Brisbane (Australia). After providing consent (and/or assent), children completed baseline assessments which were conducted by the primary investigator at the Queensland Centre for Children’s Health Research (CCHR) in Brisbane, Australia. After baseline testing, children were randomized to the therapeutic exercise program or the wait list control condition. A permuted block randomization method was used for allocation, with four children in each block. An external statistician not actively involved in the recruitment or data collection generated the randomization sequence. The allocation sequence was concealed in a set of sealed opaque envelopes labeled with the participant number. Children randomized to the exercise group started the program 1 week after baseline assessments and completed the post-program assessments within 1 week of finishing the last exercise session. The baseline and post-test assessments and delivery of the exercise program was conducted by the principal investigator (BJ); thus, assessor blinding was not possible. Participants also were not blinded to group allocation as this is not possible in an exercise intervention study. Children randomized to the control group received the intervention after completing post-program assessments.

### Therapeutic exercise program

The therapeutic exercise program aimed to improve FMS and cardiorespiratory fitness. The program comprised a combination of supervised and unsupervised exercise. The supervised sessions consisted of one 60-min session per week, conducted over 7-weeks, led by a clinical exercise physiologist (BJ). To supplement each supervised session, children completed a home-based program 2 days per week which consisted of two games from their most recent hourly session. During each 60-min session, children rotated through six activity stations where they completed developmentally appropriate games targeting specific FMS. A description of each game is provided in Table 1. The activity stations were sequenced so that a high intensity game was always followed by a low intensity game. Depending on the age of the participant, two to three new games were introduced each session. Activities were tailored to the child’s fitness and skill level by modifying the intensity and duration of each session, and/or modifying the equipment. All sessions started with a 5-min warm up and finished with a 5-min cool down consisting of stretching and relaxation exercises. Exercise intensity during each program session was monitored continuously with a heart rate monitor (PolarV800; Polar Electro Oy, Kempele, Finland). To accommodate families who had limited time and resources for travel to a research facility, participants were given the option of completing the supervised sessions at the CCHR or at home. Approximately half (52%) of the exercise sessions were completed at the CCHR, with the remaining half (48%) completed at the participant’s home.

**TABLE 1:** Outline of games played at each session of the exercise intervention.

KICKING	
Clean up the yard	The aim is to empty your yard by kicking all the balls into the neighbor’s garden. The neighbor does the same. Run time is about 5 min.
Deliver the mail	Children have to deliver (dribble and kick) mail (balls) to different houses (color marked stations).
Obstacle bowling	Instead of rolling, children kick the ball to throw over the pins/hurdles
Mr. Potato Head	Mr. Potato Head is spread over the floor but Andy is just about to come back. Therefore, the child has to kick all parts of Mr. Potato Head (different sized balls) into the toy box (goal) before Andy opens the door (a certain amount of time depending on the child’s skill level).
STRENGTH	
Help the miners	Children help the miners to get boulders out of the mine. They need to squat down to pick up a medicine ball (different weights) and carry it through a obstacle course where they have to go under and step over obstacles with the med ball in their hand.
Medicine ball boccia	Children throw (chest pass) medicine balls as close as possible to a bean bag. Receive points for how close it gets to the bean bag.
Spell the spell	They need to steal a spell from a wizard to save their friend. The children sneak into the wizard’s house, and then can grab one letter of the spell (each round is a strength circuit with different challenges like hopping in a bag, pushing med balls away, frog jumps).
Save the zoo	The animals at the zoo ran away and the child has to get them back. The child can just move as the animal that he/she wants to save!
BATTING	
Save the bird eggs	Little hurdles (40 × 20 cm) set up in a certain order. Child needs to shoot the ball with a hockey bat under the hurdles to the end and then hit the egg (ball) from a tee back in the tree (certain marked area).
Save princess peach’s castle	Child has a certain area along the wall (= castle) that he/she has to protect with the bat. Instructor rolls bombs (balls) toward the castle and the child has to bat them back.
Jewel thief	Child has to bat the golden ball far away from the goblin’s house to distract the goblin (instructor). While the goblin runs after the ball the child runs to the house and gets one of his jewels back
THROWING AND CATCHING	
Bringing home your shopping	Child helps to get the shopping (different sized balls) from the car to the fridge. Everybody is allowed to move only from one hoop to the next, and it is so hot that you can only move without shopping in your hands. Therefore, you need to throw the ball back and forth to be able to move toward the fridge.
Saving the world from meteors	Aliens (balls) attacked the world. They are all already captured but now we want to send them back to space. First child stands on moon (hoop) and catches all the aliens. From the moon, the child throws the aliens into black holes (hoops hold up by instructor).
Color train	Three stations are set up: rolling, dribbling, catching, and throwing. Child rolls the dice to see which station to go to. For each station the child receives a bean bag. Child has 5 min to see how many bean bags they can earn.
Sitting tennis	Two fields, each 2 m^2^, are separated by a net. Child and instructor sit on their knees in each field. To make a point the child’s opponent must not catch the ball in the field. If the child throws the ball out of the field, it is a point for the opponent.
BALANCE	
River crossing	To save the piglets, the child needs to cross the river over one of the four bridges: (1) little balance beam, (2) balance beam blocks separated, (3) jump on one leg along the dots (place mats), and (4) balance along the little rocks (little inflated half domes).
Hopscotch	Child throws a bean bag, and then jumps through the hopscotch grid (made out of place mats), to retrieve the beanbag.
Lily pad leap	Child is a hungry frog and only has two lily pads to cross the pond to get food. Child crosses the pond by placing one lily pad in front of the other, stepping onto it, then picking up the lily pad behind him/her and placing it in front of him/her.
Save the minions	Minions (balls) are on the little balance beam attached to bombs (cones). Child need to walk on the balance beam, step over a cone to deactivate the bomb, and then bend over to grab the minion, and carry it back to safety.
LOCOMOTION/AGILITY/COORDINATION	
Save the piglets	Piglets are held in a high security area. Child needs to turn the alarm off (touching cones in the right order) and be fast enough to get away from the security guard to save them.
Rob the bank	Child needs to run from one end of a bridge (marked area) to the other to get his/her jewels back. Instructor rolls bombs (balls) across the bridge and child needs to jump or dodge them to make it safe from one end to the other.

### Measures

#### Parental questionnaire

Parents completed a brief questionnaire providing information about their child’s health history, duration of cough, type of cough, current medication, frequency of doctor visits during the last 12 months, and family demographics (i.e., family structure, parental age, parental smoking status, and household income).

#### Fundamental movement skills

Fundamental movement skills were assessed using the Test of Gross Motor Development 2nd Edition (TGMD-2) (14). The TGMD-2 evaluates 12 movement skills representing two dimensions of gross motor performance – locomotor and object control (14). To assess children, a skill was demonstrated once, after which the child had one practice trial and two test trials, with both test scores being recorded. Scores were based on ratings of movement process characteristics for six locomotion (i.e., run, gallop, hop, leap, horizontal jump, and slide) and six object control skills (i.e., striking a stationary ball, stationary dribble, catch, kick, throw, and roll). For each skill, the nominated process characteristics were rated as “1” (present) or “0” (absent). Ratings were summed to derive a raw score for the locomotor and object control skill dimensions. The TGMD-2 is a reliable measure of proficiency in FMS in children with test-retest reliability ranging from 0.86 to 0.96 (14).

#### Cardiorespiratory fitness

Cardiorespiratory fitness was measured using a submaximal graded treadmill test following the Balke protocol for low active children (15). For ethical and safety reasons, only children aged 6 years and older completed the test. After a 2-min familiarization period, participants walked at a speed of 5.2 km/h at 6% grade, with the grade increasing by 2% every 3 min thereafter. The test was terminated at the end of the stage in which the participant’s heart rate exceeded 170 bpm. The percentage change in heart rate (%ΔHR) from rest to the completion of the first stage of the test was used as an indicator of cardiorespiratory fitness, with a decrease in %ΔHR indicative of an endurance training effect (15).

#### Perceived competence

Perceived competence was assessed using the athletic competence subscale from Harter’s Self-Perception Profile for children (16). This subscale consists of six items measuring children’s perception of their ability to do well in sports and outdoor games. To offset children’s tendency to give socially desirable responses, the Self-Perception Profile utilizes a structured alternative response format. For each item, the child is presented with a description of two children who differ on their ability to play sport or outdoor games. The child is asked to select which of the two children he or she is more like, and to indicate if the description is “really true for me” or “sort of true for me.” An example of an item is: “Some kids wish they could be a lot better at sports BUT other kids feel they are good enough at sports.” Responses were assigned a score of 1 to 4, with a score of 1 indicating the lowest perceived competence and a score of 4 indicating the highest level of perceived competence. Scores for each item were averaged to provide an overall score for perceived competence. Children aged 4–7 years completed the pictorial scale version of the scale (17). Children aged 8–13 years completed the written version of the test (16). The perceived athletic competence subscale is a reliable measure with internal consistency ranging from 0.84 to 0.91 (18). Internal consistency in the current sample was 0.54.

#### Quality of life

The Pediatric Quality of Life Inventory (PedsQL) 4.0 was utilized to assess QoL (19). The PedsQL consists of 23 items independently completed by parents and children. The domains covered include physical functioning, emotional functioning, social functioning, and school functioning. Items address functioning over the past month and responses are recorded using a 5-point Likert scale: 0 (never a problem) to 4 (almost always a problem). This measure has demonstrated reliability and validity in a diverse sample of healthy children and pediatric patients with acute or chronic health conditions (19), and within the sample, internal consistency for the total score was 0.85 for the child report and 0.90 for the parent report.

#### Parent-proxy cough-specific quality of life

The parent-proxy cough-specific quality of life (PC-QoL-8) (20) consists of eight items, exploring how their child’s cough affected their life during the previous week. Items were scored on a 7-point Likert scale, with 1 being “all the time” and 7 “none of the time.” The internal consistency of the scale within the current sample was 0.89.

### Statistical analysis

Means, SDs and frequencies were used as descriptive statistics for the sample characteristics and study outcomes. A two-way (group × time) ANOVA was used to assess the effect of the therapeutic movement program on FMS, cardiovascular fitness perceived competence, QoL and PCQOL. Data were analyzed according to the intention to treat principle, with missing values imputed using the last observation being carried forward method (21). Cohen’s *d* was used to describe effect sizes. A Cohen’s *d* of 0.2 corresponded to a small effect size, 0.5 to a medium effect size and 0.8 to a large effect size (22). All analyses were conducted using SPSS software version 26 (IBM Corp., Armonk, NY, USA) and statistical significance was set at alpha level of 0.05.

## Results

**FIGURE 1:**
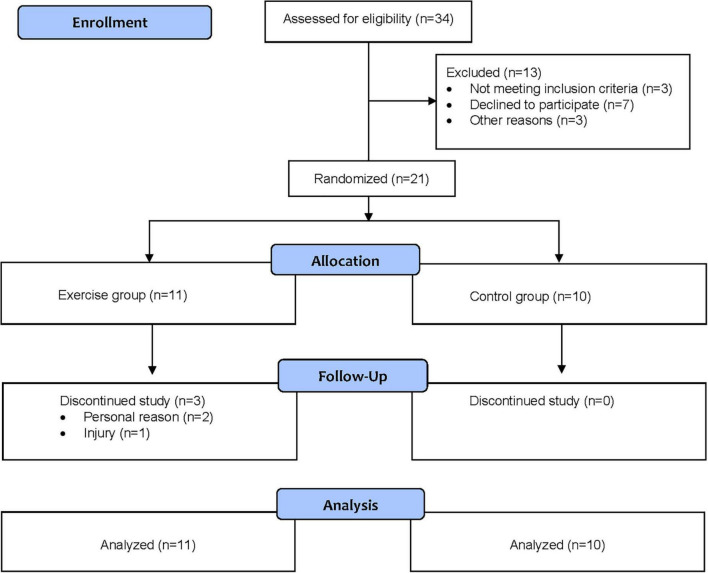
Flow of participants through the study.

In total, 21 children were enrolled in the study, with 11 randomized to the exercise group and 10 to the control group. The flow of the participants through the study is shown in Figure 1. Three children in the intervention group dropped out of the study, one due to an injury not related to participation in the exercise group, and two for personal reasons.

Descriptive statistics for the exercise group and control group are reported in Table 2. In both groups, a majority were currently on medication and reported ≥5 doctor visits in the previous year. Of the 11 children randomized to the exercise group, 8 (72.7%) completed all seven supervised exercise sessions. Of the three children who dropped out, two completed six sessions, and one completed two sessions. The mean ± SD heart rate during the supervised exercise sessions was 137 ± 12 beats per minute (bpm). On average, children’s heart rate was above 120 bpm for 81% of the session time and above 140 bpm for 47% of the session time.

**TABLE 2:** Descriptive statistics of children aged 4–13 years with non-cystic fibrosis bronchiectasis randomized to the exercise intervention (*n* = 11) or control (*n* = 10).

	Exercise (*n* = 11)	Control (*n* = 10)
Age (years)^a^	6.8 (4.3–8.8)	6.9 (5.7–8.9)
Male	73.0%	80.0%
Currently on medication^b^	8 (72.7)	6 (60.0)
Inhaled steroids	1 (9.1)	3 (30.0)
Bronchodilators	1 (9.1)	2 (20.0)
Oral steroids	0 (0.0)	1 (10.0)
Antibiotics	6 (54.5)	5 (50.0)
**Number of doctor visits in the last 12 months**		
<5 visits	18.2%	10.0%
5–9 visits	54.5%	60.0%
10–14 visits	18.2%	20.0%
15–20 visits	9.1%	10.0%
Children with a single parent	9.1%	20.0%
**Average household income^c^**		
<$25,000 AUD	0.0%	30.0%
$26,000–$50,000 AUD	18.2%	20.0%
$51,000–$75,000 AUD	9.1%	0.0%
>$76,000 AUD	72.7%	50.0%
Families with a smoker	18.2%	30.0%

AUD, Australian dollar.

^a^Data are expressed as median (interquartile range).

^b^Data are expressed as n and percentage. Some children were on more than 1 medication so percentage exceeds 100%.

^c^Poverty line <$46,551 AUD/year (37).

Table 3 presents baseline and post-test scores for object control skills, locomotor skills, perceived competence, PedsQL, and PCQoL. There was a significant group by time interaction for both locomotor (*F*_(1,18)_ = 7.6, *p* = 0.01) and object control skills (*F*_(1,18)_ = 8.3, *p* = 0.01). Children randomized to the exercise group exhibited statistically significant and substantial improvements in both locomotion and object control movement skills, while the control group exhibited little change. The associated effect sizes indicated that the exercise group had a large positive effect on locomotor and object control skills. No significant group by time interactions were observed for scores on the Harter’s perceived athletic competence scale, PedsQL and PCQoL.

**TABLE 3:** Pre- and post-test scores for fundamental movement skills, perceived competence, and health-related quality of life.

	Exercise		Control					
						
	Mean	SEM	Mean	SEM	df	*F*	*P*-value	ES
**FMS**								
Locomotor pre	29.0	2.0	31.6	2.1				
Locomotor post	35.2	2.2	31.8	2.3	1,19	7.6	0.01	1.2
Object control pre	27.0	2.0	31.0	2.1				
Object control post	35.5	2.2	32.3	2.3	1,19	8.3	0.01	1.3
**Perceived competence**								
PAC pre	3.0	0.2	3.0	0.2				
PAC post	2.8	0.2	3.0	0.2	1,19	0.2	0.63	0.2
**Quality of life**								
PQL physical pre	74.1	5.6	67.2	5.6				
PQL physical post	78.4	4.7	72.7	4.7	1,19	0.7	0.80	0.4
PQL physical pre	74.1	5.6	67.2	5.6				
PQL physical post	78.4	4.7	72.7	4.7	1,19	0.7	0.80	0.4
PQL emotion pre	73.0	4.8	64.0	4.8				
PQL emotion post	70.0	5.9	65.5	5.6	1,19	0.5	0.47	0.3
PQL social pre	73.5	6.1	70.0	6.1				
PQL social post	70.5	7.1	68.0	7.0	1,19	0.04	0.84	0.1
PQL school pre	76.0	5.3	59.5	5.3				
PQL school post	75.0	5.1	63.0	5.6	1,19	1.2	0.28	0.5
PQL total pre	74.1	4.3	65.4	4.3				
PQL total post	74.1	4.8	68.1	4.8	1,19	1.4	0.25	0.5
PC-QoL-8 pre	4.5	0.4	4.2	0.4				
PC-QoL-8 post	4.5	0.5	4.6	0.5	1,19	0.4	0.54	0.3

SEM, standard error of measurement; ES, effect size; PAC, perceived athletic competence; PQL, PedsQL 4.0 quality of life; PCQoL-8, parent-proxy cough-specific quality of life-8.

**FIGURE 2:**
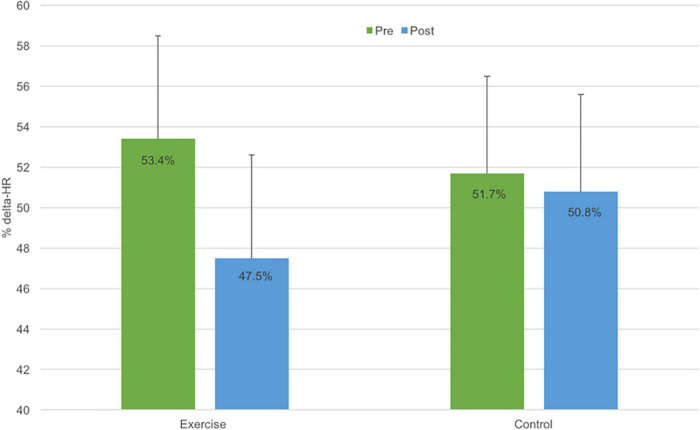
Cardiovascular fitness (expressed as % change in heart rate) at pre- (green bar) and post-exercise intervention (blue bar) for the exercise intervention (*N* = 7) and wait-list control groups (*N* = 8). Error bars represent ± standard error of measurement (SEM).

Figure 2 illustrates the effects of the exercise group on cardiorespiratory fitness. Among children completing the exercise program, %ΔHR declined 6% points while %ΔHR declined only marginally among control participants (0.9% points). However, due to the small sample size, the group by time interaction was not statistically significant (*F*_(1,13)_ = 0.9, *p* = 0.4). Expressed as an effect size, the exercise program had a moderate positive effect on cardiorespiratory fitness (Cohen’s *d* = 0.5).

## Discussion

This pilot RCT evaluated the efficacy of a therapeutic exercise program on FMS, cardiovascular fitness, perceived competence and QoL in children with bronchiectasis. The program significantly improved proficiency in FMS, with a 21% increase in locomotion skills and a 31% increase in object control. The effect sizes associated with these improvements were, by convention, large. In addition, the program had a moderately positive effect on cardiorespiratory fitness. Among children completing the exercise program, %ΔHR declined by 6% points, while children in the control group exhibited minimal change. Although the small sample size precluded statistical significance, the observed effect size for %ΔHR (*d* = 0.5) was commensurate with that reported for improvements in aerobic fitness in asthmatic children completing exercise training (23). The program had no significant impact on perceived competence or QoL. These results indicate that a play-based therapeutic exercise program can improve FMS proficiency and fitness in children with bronchiectasis and that a larger trial is warranted to test the efficacy of the program.

Previous studies have shown exercise training to be effective in improving cardiorespiratory fitness and QoL in children with asthma and CF (11, 24–26). However, to our knowledge, this is the first study to evaluate the effects of a therapeutic exercise program on proficiency in FMS in children with chronic respiratory disease. Improvements in FMS resulting from the seven-session program were not only statistically significant, but clinically important. Improving FMS is important as such improvements have been shown to contribute positively to children’s health and well-being. Children with FMS proficiency have higher levels of cardiorespiratory fitness (27, 28), perceived competence (29, 30) and self-esteem (31). Additionally, children with FMS proficiency are more likely to be physically active (32, 33) and less likely to be overweight and obese (34). Thus, by improving FMS, the capacity for children with bronchiectasis to engage in sport and other physical activities is potentially enhanced, which may mitigate the risk of disabling secondary conditions such as obesity and depression. In support of this assertion, we had undertaken a cross-sectional study which found that children with bronchiectasis who achieve their age equivalency for FMS have higher levels of physical activity, greater perceived competence, and better health-related QoL (10). Future trials evaluating the effectiveness of programs to improve FMS in children with chronic respiratory conditions such as bronchiectasis should therefore include a follow-up period of 6–12 months to determine the extent to which improvements in FMS lead to increases in physical activity performance and improvements in health outcomes such as weight status, cardiovascular fitness, and lung function.

Quality of life is an important multi-dimensional health outcome in children with chronic health conditions (19). In a systematic review and meta-analysis, it was concluded that exercise training significantly improves QoL in children with asthma and CF (26). However, our seven-session therapeutic movement program did not have a significant impact on perceived competence or QoL. This may be due to our small sample size, but other reasons include insufficient duration and intensity of our program to change these outcomes and a lag in time is required before one would expect to see improvements in QoL.

The current study had several strengths. It is the first study to examine the effects of a therapeutic movement program to improve FMS proficiency in children with bronchiectasis. Further, the therapeutic exercise program was game-based and designed to match the naturally intermittent activity patterns of children (35). Finally, the program is highly scalable. It does not require expensive equipment and can be delivered in variety of settings, including the participant’s home.

There were, however, a number of limitations that need to be considered. This was a pilot study providing initial data on the effects of a therapeutic exercise program. We therefore recruited a relatively small number of participants, resulting in low statistical power for some of the study outcomes. However, the findings are promising and provide empirical estimates for effect size and variance of the primary outcome measure for larger trials testing the efficacy of the program on FMS proficiency. Second, due to the nature of exercise intervention studies, it is not possible to blind participants to group allocation, and in this trial, assessor blinding also was not possible as the principal investigator (BJ) delivered the program and completed the assessments. While assessor bias cannot be ruled out, it is important to note that the assessments of cardiorespiratory fitness, perceived competence and QoL were instrument based and therefore did not require interpretation from the assessor. While locomotion and object control scores were based on assessor ratings, the TGMD-2 assessment protocol mitigates against observer bias by clearly delineating the critical features for each skill, which are coded as being present or absent. Third, the exercise program comprised of seven supervised 60-min weekly sessions, with a supplementary home-based program completed twice a week, which is a relatively low dose for an exercise intervention (36). Despite eliciting large positive improvements in FMS proficiency, a longer program may be required to favorably impact perceived competence and QoL. Fourth, despite randomization, there was an imbalance between the two groups in relation to the proportion of participants with a household income of greater than $76,000 AUD and families with a smoker. However, in absolute terms, the differences equated to just 1 or 2 participants per group. Fifth, as noted above, the internal consistency for Harter’s athletic competence subscale was low, potentially indicating problems with children’s understanding of the structured alternative format scale. Thus, the findings related to perceived competence need to be interpreted with considerable caution. Sixth, compliance to the home exercise component was not adequately monitored. At each supervised session, children would verbally self-report their homework from the previous week, but no data were recorded. Therefore, the full dose of the program is unknown.

## Conclusion

In conclusion, this pilot RCT provides preliminary evidence for the efficacy of a seven-session play-based therapeutic movement program to improve proficiency in FMS in children with bronchiectasis. The program had a moderate positive effect on cardiorespiratory fitness, but no improvements were noted for perceived competence and QoL. The results are sufficiently positive to warrant conducting a larger RCT testing the efficacy of the exercise program in children with bronchiectasis and/or other chronic respiratory conditions. Based on the observed effect sizes for the impact of the program on locomotor skills (Cohen *d* = 1.2) and object control skills (Cohen *d* = 1.3) and an estimated SD of 6.8 for locomotor and object control standard scores (based on the observed SD and the recommended inflation factor of 1.099), a sample size of 24 participants will be required. To offset a projected dropout rate of up to 25%, the larger trial should recruit and enroll 15 participants per group, providing a total trial sample size of *N* = 30. This calculation assumes a power level of 80% and a two-sided alpha level of 0.05. In addition, a future RCT should consider implementing the program over a longer duration (8–10-weeks) and employ accelerometer-based motion sensors to monitor the supplemental home-based exercise sessions. Where possible, future studies should be carefully designed to minimize bias (e.g., blinded assessors), and include a follow-up period to determine the extent to which improvements in FMS proficiency are maintained over time and contribute to positive changes in other health outcomes such as physical activity performance, cardiorespiratory fitness, and health-related QoL.

## Data availability statement

The raw data supporting the conclusions of this article will be made available by the authors, without undue reservation.

## Ethics statement

The studies involving human participants were reviewed and approved by the Children’s Health Queensland Hospital and Health Service (HREC/14/QRCH/136) and the Human Research Ethics Committee at the University of Queensland (2014001176). Written informed consent to participate in this study was provided by the participants’ legal guardian/next of kin.

## Author contributions

BJ and SGT designed the study. BJ collected and analyzed the data and wrote the manuscript. SGT was a major contributor in designing the study, analyzing data, and writing the manuscript. SG and ST helped designing the study and writing the manuscript. HP and AC helped with recruiting the participants and writing the manuscript. All authors read and approved the final manuscript.

## References

[1] Wurzel DF Chang AB. An update on pediatric bronchiectasis. *Expert Rev Respir Med.* (2017) 11:517–32. 10.1080/17476348.2017.1335197 28540765

[2] McCallum GB Binks MJ. The epidemiology of chronic suppurative lung disease and bronchiectasis in children and adolescents. *Front Pediatr.* (2017) 5:27. 10.3389/fped.2017.00027 28265556 PMC5316980

[3] Chang AB Bell SC Torzillo PJ King PT Maguire GP Byrnes CA Chronic suppurative lung disease and bronchiectasis in children and adults in Australia and New Zealand thoracic society of Australia and New Zealand guidelines. *Med J Aust.* (2015) 202:21–3. 10.5694/mja14.00287 25669469

[4] Kapur N Masters IB Chang AB. Exacerbations in noncystic fibrosis bronchiectasis: clinical features and investigations. *Respir Med.* (2009) 103:1681–7. 10.1016/j.rmed.2009.05.007 19501498

[5] Kapur N Masters IB Newcombe P Chang AB. The burden of disease in pediatric non-cystic fibrosis bronchiectasis. *Chest.* (2012) 141:1018–24. 10.1378/chest.11-0679 21885727

[6] Chang AB Masel JP Boyce NC Wheaton G Torzillo PJ. Non-CF bronchiectasis: clinical and HRCT evaluation. *Pediatr Pulmonol.* (2003) 35:477–83. 10.1002/ppul.10289 12746947

[7] Chang AB Fortescue R Grimwood K Alexopoulou E Bell L Boyd J European respiratory society guidelines for the management of children and adolescents with bronchiectasis. *Eur Respir J.* (2021) 58:2002990. 10.1183/13993003.02990-2020 33542057

[8] Ucgun H Gurses HN Kaya M Cakir E. Video game-based exercise in children and adolescents with non-cystic fibrosis bronchiectasis: a randomized comparative study of aerobic and breathing exercises. *Pediatr Pulmonol.* (2022) 57:2207–17. 10.1002/ppul.26026 35669989

[9] Joschtel B Gomersall SR Tweedy S Petsky H Chang AB Trost SG. Objectively measured physical activity and sedentary behaviour in children with bronchiectasis: a cross-sectional study. *BMC Pulm Med.* (2019) 19:7. 10.1186/s12890-018-0772-8 30621677 PMC6323769

[10] Joschtel B Gomersall SR Tweedy S Petsky H Chang AB Trost SG. Fundamental movement skill proficiency and objectively measured physical activity in children with bronchiectasis: a cross-sectional study. *BMC Pulm Med.* (2021) 21:269. 10.1186/s12890-021-01637-w 34404362 PMC8371810

[11] Joschtel B Gomersall SR Tweedy S Petsky H Chang AB Trost SG. Effects of exercise training on physical and psychosocial health in children with chronic respiratory disease: a systematic review and meta-analysis. *BMJ Open Sport Exerc Med.* (2018) 4:e000409. 10.1136/bmjsem-2018-000409 30305925 PMC6173241

[12] Whitehead AL Julious SA Cooper CL Campbell MJ. Estimating the sample size for a pilot randomised trial to minimise the overall trial sample size for the external pilot and main trial for a continuous outcome variable. *Stat Methods Med Res.* (2016) 25:1057–73. 10.1177/0962280215588241 26092476 PMC4876429

[13] Morgan PJ Barnett LM Cliff DP Okely AD Scott HA Cohen KE Fundamental movement skill interventions in youth: a systematic review and meta-analysis. *Pediatrics.* (2013) 132:e1361–83. 10.1542/peds.2013-1167 24167179

[14] Ulich D. *TGMD-2 test of Gross Motor Development.* Austin, TX: pro-ed (2000).

[15] Balke B Ware RW. An experimental study of physical fitness of Air Force personnel. *U S Armed Forces Med J.* (1959) 10:675–88. 10.21236/ADA036235 13659732

[16] Harter S. *Manual for the Self-Perception Profile for Children.* Denver, CO: University of Denver (1985).

[17] Harter S Pike R. *The Pictorial Scale of Percieved Competence and Social Acceptance for Young Children.* Denver, CO: University of Denver (1983). 6525886

[18] Harter S. The perceived competence scale for children. *Child Dev.* (1982) 53:87–97. 10.2307/1129640 6525886

[19] Varni JW Seid M Kurtin PS. PedsQL™ 4.0: reliability and validity of the pediatric quality of life inventory™ Version 4.0 generic core scales in healthy and patient populations. *Med Care.* (2001) 39:800–12. 10.1097/00005650-200108000-00006 11468499

[20] Newcombe PA Sheffield JK Juniper EF Petsky HL Willis C Chang AB. Validation of a parent-proxy quality of life questionnaire for paediatric chronic cough (PC-QOL). *Thorax.* (2010) 65:819–23. 10.1136/thx.2009.133868 20805179

[21] Armijo-Olivo S Warren S Magee D. Intention to treat analysis, compliance, drop-outs and how to deal with missing data in clinical research: a review. *Phys Ther Rev.* (2009) 14:36–49. 10.1179/174328809X405928

[22] Cohen J. *Statistical Power Analysis for the Behavioural Sciences.* 2nd ed. Hillsdale, NJ: Lawrence Erlbaum Associates (1988).

[23] van Veldhoven NH Vermeer A Bogaard JM Hessels MG Wijnroks L Colland VT Children with asthma and physical exercise: effects of an exercise programme. *Clin Rehabil.* (2001) 15:360–70. 10.1191/026921501678310162 11518437

[24] van Doorn N. Exercise programs for children with cystic fibrosis: a systematic review of randomized controlled trials. *Disabil Rehabil.* (2010) 32:41–9. 10.3109/09638280902991842 19925275

[25] Wanrooij VH Willeboordse M Dompeling E van de Kant KD. Exercise training in children with asthma: a systematic review. *Br J Sports Med.* (2014) 48:1024–31. 10.1136/bjsports-2012-091347 23525551

[26] Crosbie A. The effect of physical training in children with asthma on pulmonary function, aerobic capacity and health-related quality of life: a systematic review of randomized control trials. *Pediatr Exerc Sci.* (2012) 24:472–89. 10.1123/pes.24.3.472 22971562

[27] Lubans DR Morgan PJ Cliff DP Barnett LM Okely AD. Fundamental movement skills in children and adolescents: review of associated health benefits. *Sports Med.* (2010) 40:1019–35. 10.2165/11536850-000000000-00000 21058749

[28] Okely AD Booth ML Patterson JW. Relationship of cardiorespiratory endurance to fundamental movement skill proficiency among adolescents. *Pediatr Exerc Sci.* (2001) 13:380–91. 10.1123/pes.13.4.380

[29] Barnett LM Morgan PJ van Beurden E Beard JR. Perceived sports competence mediates the relationship between childhood motor skill proficiency and adolescent physical activity and fitness: a longitudinal assessment. *Int J Behav Nutr Phys Act.* (2008) 5:40. 10.1186/1479-5868-5-40 18687148 PMC2569960

[30] Rudisill ME Mahar MT Meaney KS. The relationship between children’s perceived and actual motor competence. *Percept Mot Skills.* (1993) 76:895–906. 10.2466/pms.1993.76.3.895 8321605

[31] Martinek TJ Cheffers JT Zaichkowsky LD. Physical activity, motor development and self-concept: race and age differences. *Percept Mot Skills.* (1978) 46:147–54. 10.2466/pms.1978.46.1.147 643470

[32] Okely AD Booth ML Patterson JW. Relationship of physical activity to fundamental movement skills among adolescents. *Med Sci Sports Exerc.* (2001) 33:1899–904. 10.1097/00005768-200111000-00015 11689741

[33] Williams HG Pfeiffer KA O’neill JR Dowda M McIver KL Brown WH Motor skill performance and physical activity in preschool children. *Obesity.* (2008) 16:1421–6. 10.1038/oby.2008.214 18388895

[34] Okely AD Booth ML Chey T. Relationships between body composition and fundamental movement skills among children and adolescents. *Res Q Exerc Sport.* (2004) 75:238–47. 10.1080/02701367.2004.10609157 15487288

[35] Bailey RC Olson J Pepper SL Porszasz J Barstow TJ Cooper DM. The level and tempo of children’s physical activities: an observational study. *Med Sci Sports Exerc.* (1995) 27:1033–41. 10.1249/00005768-199507000-00012 7564970

[36] Ram FS Robinson SM Black PN. Effects of physical training in asthma: a systematic review. *Br J Sports Med.* (2000) 34:162–7. 10854014 10.1136/bjsm.34.3.162 PMC1763260

[37] Davidson P Saunders P Bradbury B Wong M. *Poverty in Australia 2020. Part 1 Overview. ACOSS/UNSW Poverty and Inequality Partnership Report.* Sydney, NSW: ACOSS (2020).

